# Health Status of Tsimihety Women: Sexually Transmitted Infections and Schistosomiasis, Northern Madagascar

**DOI:** 10.3390/jcm14103479

**Published:** 2025-05-16

**Authors:** Daniel Kasprowicz, Wanesa Wilczyńska, Krzysztof Korzeniewski

**Affiliations:** 1 Clinique Medicale Beyzym, Manerinerina, Ambatoboeny District, Manerinerina 403, Madagascar; daniel.kasprowicz@icloud.com; 2 Department of Epidemiology and Tropical Medicine, Military Institute of Medicine—National Research Institute, 04-141 Warsaw, Poland; wwilczynska@wim.mil.pl

**Keywords:** STIs, schistosomiasis, women, Madagascar

## Abstract

**Background**: Madagascar is one of the lowest-income countries in Africa, and it has a poorly developed healthcare system. Malagasy women face limited access to sexual and reproductive health services, which is a serious risk factor facilitating the spread of sexually transmitted infections (STIs). The aim of the present study was to assess the prevalence of STIs (*Trichomonas vaginalis*, *Neisseria gonorrhoeae*, *Treponema pallidum*, and HIV-1/HIV-2) and urogenital schistosomiasis, as well as to evaluate hematological parameters and nutritional status, in a group of women from northern Madagascar. **Methods**: The study was conducted in April 2024 at the Clinique Médicale Beyzym in Manerinerina, Ambatoboeny District. Samples, which included overnight urine, venous blood, and vaginal swabs, were collected from 159 women aged 15–80 years. The urine samples were examined for the presence of *Schistosoma haematobium* eggs by light microscopy, the vaginal swabs were tested for the presence of *Trichomonas vaginalis* and *Neisseria gonorrhoeae* infections (by light microscopy), and venous blood samples were collected into VACUTAINER SEC collection tubes without anticoagulant and were tested for HIV-1/HIV-2 and *Treponema pallidum* infections using test cassettes. **Results**: The prevalence of STIs in the study group was found to be 31.5%, while *S. haematobium* infections were found in 17.6% of the tested women. Cases of gonorrhea (20.1%), trichomoniasis (8.8%), syphilis (7.6%), and one case of HIV infection were identified. **Conclusions**: The study found a high prevalence of STIs and *S. haematobium* cases in Tsimihety women. In order to improve the quality of healthcare in Madagascar, it is necessary to improve accessibility to maternal, sexual, and reproductive health services.

## 1. Introduction

Madagascar is one of the lowest-income countries in Africa, with a poverty rate of over 80% [1]. The country’s healthcare sector is seriously underfunded, and the healthcare expenditure per capita is insufficient to guarantee even the most basic level of healthcare to all citizens. It is estimated that nearly 26% of Malagasy people live more than 10 km from the nearest healthcare facility, less than 10% have comprehensive health insurance, and nearly 50% of healthcare facilities in Madagascar are not accessible year-round [1,2]. The healthcare sector in Madagascar, which is mostly funded by external sources (the private sector or household expenditures), faces many challenges. One of these challenges is a serious shortage of medical personnel, but there are many more, for example, a lack of access to reliable electricity sources and safe water sources in many parts of the country. All these factors are associated with growing levels of unmet healthcare needs in the Malagasy population, mostly resulting from limitations in diagnosis, treatment, and the effective prevention of many diseases [3]. In Madagascar, the major public health concerns are outbreaks of infectious diseases, parasitic illnesses (including schistosomiasis), malnutrition, non-infectious diseases, and high maternal and neonatal mortality (which are mostly due to complications in home birth or a lack of assistance from a qualified midwife at birth) [3,4]. In fact, there is a great scarcity of maternal and neonatal care centers across the country. Also, Malagasy women are constrained in seeking sexual and reproductive advice by limited access to healthcare services dedicated to women, a lack of reliable sources of information, and the prohibitive costs of consultations. Many Malagasy women marry at a young age (18% of women report having had their first sexual intercourse before the age of 15, while 31.1% get pregnant with their first child between the ages of 15 and 19 years old). Despite their young age, almost 72% of Malagasy women claim they make their own decisions regarding their sexual relations, the use of contraceptives, and reproductive health [5]. The scarcity of healthcare facilities and social stigma remain significant barriers to accessing professional sexual and reproductive healthcare for many women living in Madagascar [6]. Despite the presence of elements that favor low rates of STIs—including the insular position of Madagascar, its low level of migration, and efficient prevention programs that seem to constitute protective factors against STI transmission—the prevalence of STIs in the country is in fact high [7,8].

The aim of the present study was to assess the prevalence of STIs (*Trichomonas vaginalis*, *Neisseria gonorrhoeae*, *Treponema pallidum*, and HIV-1/HIV-2) and urogenital schistosomiasis, as well as to evaluate hematological parameters and nutritional status, in a population of women residing in Ambatoboeny District, northern Madagascar.

## 2. Materials and Methods

### 2.1. Study Group and Sample Collection

The study was conducted in April 2024 at the Clinique Médicale Beyzym in Manerinerina (a rural municipality), Ambatoboeny District, Boeny Region, northern Madagascar (16°17′ S 47°17′ E). Samples were collected from 159 women aged 15–80 living in Manerinerina and surrounding villages during the second half of the rainy season. The samples included the following: (1) overnight urine, (2) venous blood, and (3) vaginal swabs. Additionally, basic anthropometric parameters of each participant were taken: body weight, height, and Body Mass Index (BMI). Each participant completed a questionnaire and provided consent to participate in the study in both French and Malagasy. For illiterate women, the procedure was explained by qualified Malagasy medical personnel.

### 2.2. Sociocultural and Economic Barriers to Healthcare Access

In the Tsimihety community, access to healthcare is significantly hindered by both economic and sociocultural factors. A substantial portion of the Malagasy population lives below the international poverty line, with approximately 75% subsisting on less than USD 1.90 per day. This economic hardship often leads individuals to forgo medical consultations, diagnostics, or treatments due to prohibitive costs. Moreover, gender dynamics within the community further restrict women’s access to healthcare. Women are frequently financially dependent on male partners, who may withhold consent for them to seek medical care. This dependency is compounded by cultural norms that stigmatize sexually transmitted infections (STIs), often attributing blame to women, despite observations indicating that men are more likely to have multiple sexual partners. Such stigma discourages women from seeking necessary medical attention for STIs [9,10,11].

Additionally, traditional beliefs and practices influence healthcare-seeking behavior. Many community members attribute illnesses to spiritual or ancestral causes, leading them to consult traditional healers instead of medical professionals [12]. This preference for traditional medicine can delay or prevent access to effective biomedical treatments [4].

These findings underscore the need for culturally sensitive health interventions that address economic barriers, empower women, and integrate traditional beliefs with biomedical practices to improve healthcare access in the Tsimihety community.

#### 2.2.1. Light Microscopy

##### Urine Sediment Examination

Urine was examined for the presence of *Schistosoma haematobium* eggs. Urine samples were collected directly after an overnight rest, using the first morning portion of urine. The urine was collected from the mid-stream following light physical exertion, in accordance with the diagnostic recommendations of the Pasteur Institute in Antananarivo (Institute Pasteur de Madagascar) [13]. Each patient was instructed on how to properly collect a 40 mL urine sample into a sterile container. Next, 10 mL of urine was centrifuged at 1500–2000 rpm for 5 min, after which, 9.5 mL of urine was removed by decantation. The sediment was then examined using direct light microscopy by a qualified laboratory diagnostician at Clinique Médicale Beyzym. One sample per participant was analyzed, and each was examined once using a qualitative approach to determine the presence or absence of *S. haematobium* eggs.

##### Vaginal Swab Examination

Vaginal swabs were analyzed for the presence of *Trichomonas vaginalis* and *Neisseria gonorrhoeae* infections. Before the sampling began, participants were informed about the purpose and procedure of the study, as well as the exclusion criteria. The sample was collected by obstetric staff using a single-use gynecological speculum and a swab without transport media. Each swab was moistened with 5 drops of sterile 0.9% NaCl (the direct analysis of the sample was performed in real time in the clinic’s laboratory using light microscopy). Simultaneously, a direct smear was prepared from the collected swab on a glass slide and subjected to Gram staining, followed by microscopic examination to detect gonococci. For every vaginal swab collected, two slides were prepared and examined under a light microscope at 400× magnification. All slides were analyzed by a single trained laboratory technician to ensure consistency in interpretation.

Prior to sample collection, participants received detailed instructions regarding the nature and purpose of the examination. A brief interview was conducted to assess recent use of antibiotics, antiparasitic medications, or other treatments that might interfere with diagnostic results.

Exclusion criteria included menstruation at the time of examination; antibiotic or antiparasitic use within 14 days prior to sampling; vaginal douching within 48 h; sexual intercourse within the preceding 7 days; genital bleeding unrelated to menstruation; and inability to provide informed consent.

#### 2.2.2. Measurement of Hematological Parameters

Venous blood was collected into a VACUTAINER EDTA-K3 tube (Fisher Scientific, Göteborg, Sweden) by qualified personnel. Hematological parameters were measured using a fully automated Mindray BC 3000 Plus analyzer. Reference values for non-pregnant women were based on World Health Organization (WHO) guidelines.

The inclusion of hematological parameters in this study was grounded in the theoretical framework of syndemics, which highlights the interaction between biological and socio-environmental factors that exacerbate disease burden in disadvantaged communities. Nutritional status and hematological health can influence both susceptibility to and the outcomes of infections, particularly in the context of co-occurring parasitic and sexually transmitted diseases.

#### 2.2.3. Immunochromatographic Tests

Venous blood was collected from all participants into a VACUTAINER SEC tube without anticoagulant. After collection, the blood was allowed to stand for approximately 30–60 min. A 5 min centrifugation was performed at 4000 rpm, and the resulting serum was transferred to a single-use round-bottom tube. Testing for HIV-1/HIV-2 infection and *Treponema pallidum* (TP) infection was conducted using immunochromatographic techniques. For syphilis testing, we used Cypress Diagnostics Syphilis Ab Rapid C cassette tests. For HIV screening, two tests were employed: the Cypress Diagnostics HIV ½ and the DETERMINE^TM^ HIV-1/2 rapid tests. All of these are direct binding tests designed for the qualitative detection of antibodies against HIV-1/HIV-2 and TP (and antigen, in the case of DETERMINE^TM^) in whole blood, serum, or plasma.

We used tests that have been validated by the manufacturer and in independent studies. The Cypress Diagnostics Syphilis Ab Rapid C test showed a relative sensitivity of 98.7% and a specificity of 98.8% in a blind validation study involving 155 clinical samples. The Cypress Diagnostics HIV 1/2 test has demonstrated sensitivity and specificity values exceeding 99%, according to manufacturer data. The DETERMINE™ HIV-1/2 test has shown 100% sensitivity and a specificity of 99.23% (Ab) and 99.66% (Ag) in previously published validations.

**Figure 1:**
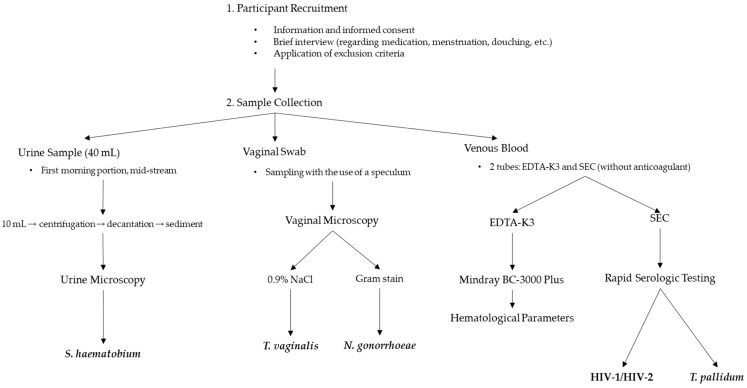
Schematic overview of the study design and diagnostic procedures.

Although no local pre-study validation was conducted due to limited laboratory infrastructure, all tests were performed strictly according to the manufacturers’ protocols by trained personnel. All selected test kits are CE-marked and are widely used in resource-limited healthcare settings (Figure 1).

### 2.3. Statistical Analysis

All statistical analyses were conducted using an Excel spreadsheet (Microsoft Corporation, Redmond, WA, USA) and Statistica, version 13 (a data analysis software system) [TIBCO Software Inc., Palo Alto, CA, USA (2017)], available at http://www.statsoft.pl (accessed on 20 December 2024). A *p*-value of 0.05 was considered the threshold for statistical significance in all analyses. For comparisons between two independent groups, Student’s t-test was used when the data met the assumptions of normality and homogeneity of variances. In cases where the normality assumption was not met, the Mann–Whitney U test was applied as a non-parametric alternative.

### 2.4. Ethical Approval

This research project was approved by the Ministry of Public Health of Madagascar in Antananarivo (No. 108-24/MSANP/SPC of 5 April 2024). Parents or guardians of children were required to provide an informed consent form for their child’s participation in the study. Additionally, informed consent was obtained from participants aged 15–17 years, ensuring that they understood the study procedures, potential risks, and benefits and that their participation was voluntary. The collection of samples was supervised by the medical personnel employed at the Clinique Medicale Beyzym from Marinerina (Ambatoboeny District, Northern Madagascar).

### 2.5. Study Variables

This study aimed to determine whether there is a correlation between the prevalence rates of STIs and the ages, levels of education, hematological profile, and nutritional statuses (which were calculated based on BMI measurements) of the study participants. The results obtained were compared with the findings of other, similar studies, which were accessed from publicly available databases.

## 3. Results

**Figure 2:**
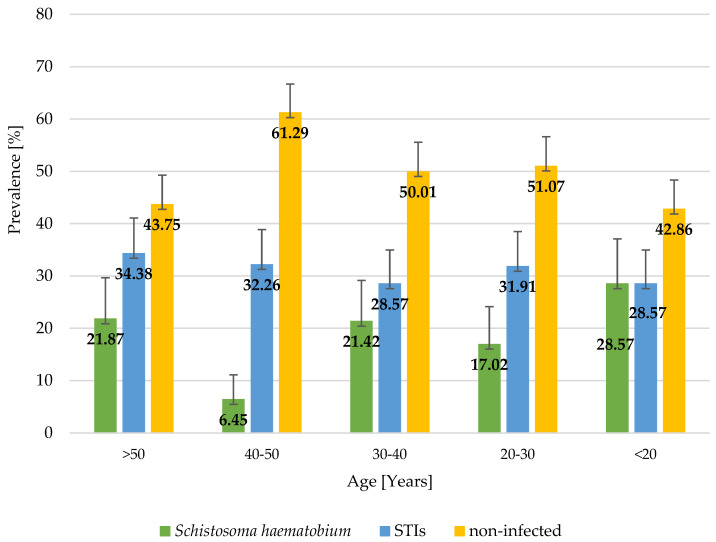
Distribution of STIs and Schistosoma haematobium in women (n = 159) by age, northern Madagascar, 2024.

A total of 159 women aged 15–80 years were involved in the study. Most of the study participants had primary education (42.1%), followed by secondary education (31.5%) and tertiary education (7%); 18.9% of the study participants had no formal education, and one person did not give an answer to the question regarding her education level. Most of the study participants had normal BMIs, 35 women were underweight, 31 were overweight, and 7 were obese (1 of these women was diagnosed with morbid obesity > 40 kg/m^2^). The analysis of the hematological parameters (hemoglobin measurements) demonstrated that 66% of the women involved in the study had anemia; 14 of the women were diagnosed with moderate anemia, and 5 with severe anemia. One in three women (31.5%) was found to have at least one STI, and 17.6% of the study participants were found to be infected with *Schistosoma haematobium*. The results showed that the most prevalent STIs included infections caused by *Neisseria gonorrhoeae* (20.1%), followed by *Trichomonas vaginalis* (8.8%) and *Treponema pallidum* (7.6%). Also, there was a single case of HIV infection. A total of 7.6% of the women included in the study were found to have co-infections, of which *T. pallidum* + *N. gonorrhoeae* co-infection was the most prevalent. The study found that STI infection rates were higher in women with hemoglobin concentrations below the reference values and in women with an abnormal BMI (40% of underweight women, 32.3% of overweight women, and 71% of women with obesity). However, those differences were not statistically significant (*p*-value > 0.05). Regarding *S. haematobium* infection rates, no correlation was found between BMI or the presence of anemia and the occurrence of this infection. The level of education was not shown to significantly affect STI rates; however, most infections were seen in women with primary education (10% more than in all other groups). The number of *S. haematobium* cases was found to decrease with a higher level of education. There were no cases of *S. haematobium* infection in women with tertiary education. The distribution of STIs and *Schistosoma haematobium* infections in women by anemia, BMI, and level of education is shown in Table 1. The prevalence of STIs was found to be similar in all age groups (around 30%), while the prevalence rate of *S. haematobium* infections differed between individual age groups, with the highest prevalence seen in the age group below 20 years and the lowest in the age group comprising women aged between 40 and 50 years. The distribution of STIs and *Schistosoma haematobium* infections by age is shown in Figure 2.

No statistically significant differences were found between the presence of STIs or *S. haematobium* infection and hematological parameters, with the exception that the mean white blood cell count (WBC) was found to be significantly higher in women with STIs. The differences in hematological parameters between infected vs. non-infected women living in northern Madagascar in 2024 are presented in Table 2.

## 4. Discussion

The socio-economic burden of sexually transmitted infections is high globally, but it is the greatest in lower-income countries, where STIs are estimated to account for 17% of economic losses due to illness [14]. The World Health Organization (WHO) has estimated that, annually, approximately 374 million new cases of curable STIs occur globally, and that Sub-Saharan Africa accounts for approximately 40% of the global STI burden [15,16].

However, little is known about the actual prevalence of sexually transmitted illnesses in the Malagasy population, despite the exceptionally high burden of STIs on the island. Women’s sexual and reproductive health is rarely addressed by local healthcare professionals, and therefore, STIs are not routinely reported to competent health authorities [17,18]. In addition, most STIs are often asymptomatic or cause mild symptoms. When symptoms do occur, however, most Malagasy women tend to treat the infection themselves rather than seek professional medical advice. This behavior can be explained by their cultural beliefs, as well as widespread social stigma related to sexual and reproductive health [6]. STIs mostly affect young adults [19,20,21] and are especially dangerous to pregnant women due to the risk of perinatal transmission of pathogens from a mother to a child, as well as an STI’s potential to cause pregnancy complications [20]. Another major obstacle for women in accessing information on sexual and reproductive health (including the methods of STI prevention) is ethical considerations. In Madagascar, the traditional roles of women are still much valued. This is a significant problem for younger women, who are at a higher risk of STI transmission due to their limited knowledge of STI-related risk factors, as well as difficulties in negotiating safe sex with their partners. However, the results of the present study did not demonstrate a higher prevalence of STIs among reproductive-age women compared to other age groups; a similar high proportion of STI-positive cases was seen in all age groups studied.

Shewarega et al. [22] conducted a research study aimed at evaluating the pooled prevalence of STI-related care-seeking behavior among reproductive-age women living in East African countries. That study, which was based on data from national health surveys conducted between 2008/09 and 2018/2019, demonstrated that only half of the surveyed women were seeking medical care for sexually transmitted infections. According to reports available in the literature, the overall prevalence of STIs in Africa varies significantly between countries, and it is estimated at 4.5% in Kenya [20], 6.3% in Tanzania [20], 7.4% in Nigeria [20], 16.7% in Ethiopia [19], 21.3% in Uganda [20], and as much as 53.6% in Gambia [23]. The actual prevalence of STIs in Madagascar is uncertain due to a lack of reporting. According to the latest reports from Madagascar, the prevalence of STIs in the general population varies between 3% and 56%, depending on the sample composition and study setting [24,25,26,27,28].

The prevalence of STIs in this study group (159 women) was found to be 31.5%, which is similar to the results obtained by Leutscher et al. [24], who studied a group of Malagasy women from rural settings in 2005 (37.5%). The present study found cases of trichomoniasis (8.8%), syphilis (7.6%), and gonorrhea (20.1%). A comparison between the results obtained in previous studies by other authors (covering the last two decades) and the results of our study showed a reduction in the number of trichomoniasis cases [25] and an increase in syphilis and gonorrhea cases [25,26,27,29]. Recent data confirm the persistence of a high STI burden in Madagascar. A 2023 cross-sectional study among 491 women aged 18–45 in Antananarivo revealed infection rates of 11.8% for *C. trachomatis*, 4.2% for *N. gonorrhoeae*, and 14.8% for *T. vaginalis* and a 39.6% prevalence of bacterial vaginosis. Notably, one-third of women with at least one STI were asymptomatic, highlighting the silent nature of many infections and the importance of routine screening and prevention strategies [30].

The present study showed no correlation between the level of education or age of women and STI infection rates. According to other authors, STI-related care-seeking behavior is higher among women with a higher educational status and among women at an older age, which is potentially associated with their higher health awareness and better access to sexual and reproductive health services [22]. Our study found no co-infections in women with tertiary education; in fact, most co-infections were seen in the group comprising women with no formal education. The overall co-infection rate in the study sample was 7.6%. The presence of one STI is itself a risk factor for acquiring another STI [30]. Early diagnosis and treatment of STIs are, therefore, key to reducing the prevalence and preventing the spread of STIs [31]. Untreated STIs can increase a person’s risk of being infected with human immunodeficiency virus (HIV) by up to fourfold [16]. The present study found a single case of HIV infection (0.6%), which is in line with the reports on HIV prevalence in Madagascar by other authors [27,28,30]. Such a low HIV prevalence in Madagascar is quite surprising since poverty and limited access to healthcare are considered to be the major risk factors contributing to the spread of HIV and other STIs. The reason for such a huge disproportion between the prevalence of HIV and the prevalence of other STIs in Madagascar is likely the effect of the significant efforts of non-governmental organizations to control HIV transmission in the region. The highest HIV prevalence can be observed among people from high-risk groups, such as sex workers, drug addicts, and homosexuals [8]. Anemia is a frequent complication of HIV infection; its occurrence is associated with the progression of the disease, as well as the use of certain chemotherapeutic agents [32]. In this study, the HIV-positive case was a woman with hemoglobin concentration of 9.2 g/dL, which supports the abovementioned findings. Our study found no correlation between the presence of anemia and other STIs. It showed that 16% of the women with a confirmed STI also had an *S*. *haematobium* infection. There are other reports in the literature suggesting frequent co-infections between schistosomiasis and STIs. The observed association between STIs and schistosomiasis may be explained by biological mechanisms such as mucosal damage and inflammation, which can facilitate the acquisition and transmission of sexually transmitted infections [33]. Inflammatory pathways play a key role in increasing STI susceptibility by disrupting epithelial barriers, attracting immune cells that can serve as targets for viral entry (such as CD4+ T cells in the case of HIV), and altering the vaginal microbiome. Chronic genital inflammation has been shown to increase the expression of adhesion molecules and pro-inflammatory cytokines (e.g., IL-1β, TNF-α, and IL-6), creating a microenvironment that promotes pathogen invasion and persistence. This is particularly relevant in female genital schistosomiasis, where egg-induced granulomatous inflammation and ulcerations of the mucosa facilitate entry points for STI pathogens. Furthermore, inflammation-mediated epithelial damage may impair the local immune response and increase the risk of co-infections [34]. Madagascar reports one of the highest rates of schistosomiasis in the world [35], with an estimated overall prevalence of 52.2% [36]. Chronic *S. haematobium* infection is particularly dangerous for women, as it may lead to female genital schistosomiasis (FGS). FGS, like many other STIs, is believed to be a serious risk factor for acquiring HIV infection, and it may also be associated with complications such as cervical cancer, ectopic pregnancy, and infertility [37,38]. The global burden of FGS is not known; however, it is estimated that FGS affects 40–56 million women worldwide [37]. According to the latest reports, the prevalence of FGS in Madagascar is over 60% [39]. The symptoms of FGS (pain, itching, and vaginal discharge) closely resemble those of other STIs, and therefore, the disease is often misdiagnosed and mistreated [40]. In addition, a study by Mazigo et al. [41] highlighted insufficient knowledge of FGS signs and symptoms both among women from endemic communities and among healthcare workers. The prevalence of *S. haematobium* infection in the present study was found to be 17.6%; no correlation was found between the presence of the infection and anemia or BMI. Most invasions were seen in the group of women with no formal education, and no cases of schistosomiasis were found in women with tertiary education, which might be attributable to their higher health awareness, better hygiene practices, and the type of work they perform. This finding suggests that higher educational attainment may have a protective effect against schistosomiasis. Higher education is often associated with better awareness of hygiene practices, which could reduce exposure to contaminated water sources, a major transmission route for *S. haematobium*. Educated individuals are also more likely to have better access to healthcare services, which could contribute to the earlier detection and treatment of infections, reducing their spread within the community. Additionally, educated individuals may be more likely to adopt behaviors that decrease the risk of schistosomiasis, such as avoiding contact with untreated water and engaging in safer agricultural or recreational practices [42,43].

Although visual inspection of age-stratified prevalence (Figure 1) suggests potential trends in infection rates across age groups, statistical analysis did not confirm a significant association between age and *S. haematobium* infection (Mann–Whitney U test, *p* = 0.889). This finding implies that, in this sample, age does not appear to be a determining factor for infection. However, this may be due to the limited sample size or the cross-sectional design of the study, which restricts the ability to infer causality or temporal patterns. Future studies with larger and more representative populations are needed to further explore possible age-related trends and their epidemiological implications.

Seasonal variation in schistosomiasis transmission should also be considered when interpreting the findings of this study. Data collection took place during the second half of the rainy season, a period typically associated with increased human contact with freshwater sources such as rivers and rice paddies. These environmental conditions may lead to a higher risk of exposure to *S. haematobium* due to the presence of infected intermediate snail hosts in stagnant or slow-moving water. As a result, the observed prevalence of schistosomiasis in this study might be higher than what would be detected during the dry season, potentially introducing seasonal bias [44].

Despite continuous efforts to improve healthcare coverage in Madagascar, many districts in the northern and central parts of the country, i.e., the regions inhabited by the Tsimihety people, face ongoing problems with healthcare access. A scarcity of healthcare facilities, shortages of qualified personnel, and difficulties in reaching the nearest healthcare provider (especially for people living in remote settings) remain the major barriers to the timely diagnosis and effective treatment of schistosomiasis and STIs. It should also be mentioned that many people from these communities distrust modern medicine and prefer traditional methods of treatment over conventional ones, which obviously is a serious obstacle to implementing effective interventions [45].

## 5. Limitations

This study has several limitations that should be acknowledged. First, the relatively small sample size (n = 159) and the focus on a single rural area in northern Madagascar limit the generalizability of the findings to the wider Malagasy population. The results should, therefore, be interpreted with caution, as they may not reflect STI and schistosomiasis prevalence or associated risk factors in other regions or urban settings. Second, the cross-sectional design does not allow for the assessment of causal relationships between infections and the studied hematological or anthropometric parameters. Third, the study was conducted during the second half of the rainy season, which may influence the prevalence of certain parasitic infections, such as *S. haematobium*, due to seasonal variation in transmission risk. The rainy season is associated with increased water availability and flooding, which can expand the habitats of intermediate snail hosts and increase the frequency of human–water contact, particularly in agricultural and domestic settings. This seasonal context may have temporarily elevated the risk of *S. haematobium* transmission during the study period, potentially leading to an overestimation of prevalence compared to drier months. Consequently, the findings may not be generalizable to periods of lower transmission risk or to populations with different seasonal exposure patterns. Future studies should consider seasonal variation when assessing the epidemiology of schistosomiasis to better understand transmission dynamics over time.

In addition, the analysis did not include key potential confounding variables such as occupation or water contact behaviors due to a lack of data. Moreover, self-reported data from questionnaires may be subject to recall bias or underreporting, particularly for sensitive topics related to sexual and reproductive health. Furthermore, the potential underreporting of sexually transmitted infections (STIs), including HIV, due to social stigma and concerns about privacy may lead to a lower reported prevalence. Stigma associated with HIV and other STIs might discourage individuals from seeking testing or disclosing their status, thus leading to the underestimation of their true prevalence. The low HIV prevalence (0.6%) observed in our study may reflect these reporting biases rather than accurately representing the true burden of HIV within the population. Finally, the detection of only a single HIV-positive case limits the study’s statistical power to examine potential associations between HIV infection and other clinical or demographic variables.

The diagnostic methods used in this study were not locally validated, which may impact the reliability of the results. The performance characteristics of laboratory tests, such as sensitivity and specificity, can vary depending on local epidemiological and environmental factors. Without local validation, there is a risk of misclassification due to false-negative or false-positive results, particularly for infections with low-intensity presentations or cross-reactivity. This limitation should be considered when interpreting the reported prevalence rates, as diagnostic accuracy directly influences estimates of disease burden and the identification of associated risk factors.

Finally, we did not apply corrections for multiple comparisons, as this study was exploratory in nature and aimed at identifying potential associations rather than confirming specific hypotheses. However, we acknowledge that the absence of multiplicity adjustments increases the risk of type I errors, and some statistically significant findings should, therefore, be interpreted with caution.

Despite these limitations, this study provides valuable epidemiological data from an underrepresented region and highlights the need for larger, population-based studies in Madagascar to inform public health strategies.

## 6. Conclusions

The present study, which involved a group of Tsimihety women, demonstrated high prevalence rates of STIs and *S. haematobium* infections in the communities living in northern Madagascar. To improve health coverage in the region, comprehensive actions are needed. It is particularly important to develop the healthcare system and improve the healthcare infrastructure (i.e., build new healthcare facilities) in the country, but it is also essential to train local medical personnel in the diagnosis and treatment of schistosomiasis and STIs. Community-based health education programs need to address the sexual and reproductive health of women (including effective STI prevention methods), but at the same time, they should take into account the cultural and social norms of the local people and the specific needs of the women from a given community. Ensuring access to contraception (condoms or other contraceptives) and close cooperation with non-governmental organizations is key to the successful implementation of control programs in countries with high STI and schistosomiasis burdens. In addition, mobile clinics and community health worker training can help improve access to care in remote areas, further supporting the fight against these infectious diseases.

## Figures and Tables

**Table 1:** Distribution of STIs and Schistosoma haematobium in women (n = 159) by anemia, BMI, and education, northern Madagascar, 2024.

Variables	Anemia			BMI					Education				
	n (%)	Hb < 12	Hb ≥ 12	Underweight (<18.5)	Normal (18.5–24.9)	Over-Weight (25–29.9)	Obese (30–34.9)	Extremely Obese (>35)	NON	CEPE	BEPC + BACC	LICENCE/M2	No Data
Total	159 (100.0)	105 (66.0)	54 (34.0)	35 (22.0)	86 (54.1)	31 (19.5)	6 (3.8)	1 (0.6)	30 (18.9)	67 (42.1)	50 (31.4)	11 (6.9)	1 (0.6)
STIs	50 (31.5)	30 (28.6)	20 (37.0)	14 (40.0)	21 (24.4)	10 (32.3)	4 (66.7)	1 (100.0)	8 (26.7)	25 (37.3)	13 (26.0)	3 (27.3)	1 (100.0)
*Trichomonas vaginalis*	14 (8.8)	10 (9.5)	4 (7.4)	4 (11.4)	5 (5.8)	4 (12.9)	1 (16.7)	0 (0.0)	2 (6.7)	5 (7.5)	6 (12.0)	0 (0.0)	1 (100.0)
*Treponema pallidum*	12 (7.6)	8 (7.6)	5 (7.4)	8 (22.9)	3 (3.5)	0 (0.0)	1 (16.7)	0 (0.0)	5 (16.7)	6 (9.0)	0 (0.0)	1 (9.1)	0 (0.0)
*Neisseria gonorrhoeae*	32 (20.1)	17 (16.2)	15 (27.8)	6 (17.1)	16 (18.6)	7 (22.6)	2 (33.3)	1 (100.0)	3 (10.0)	19 (28.4)	8 (16.0)	2 (18.2)	0 (0.0)
HIV	1 (0.6)	1 (0.9)	0 (0.0)	0 (0.0)	1 (1.2)	0 (0.0)	0 (0.0)	0 (0.0)	0 (0.0)	0 (0.0)	1 (2.0)	0 (0.0)	0 (0.0)
*Schistosoma haematobium*	28 (17.6)	19 (18.1)	9 (16.7)	5 (14.3)	17 (19.8)	5 (16.1)	1 (16.7)	0 (0.0)	12 (40.0)	13 (19.4)	3 (6.0)	0 (0.0)	0 (0.0)
Co-infections	12 (7.6)	8 (7.6)	4 (7.4)	4 (11.4)	5 (5.8)	2 (6.5)	1 (16.7)	0 (0.0)	4 (13.3)	5 (7.5)	3 (6.0)	0 (0.0)	0 (0.0)
*S. haematobium* + *T. vaginalis*	2 (1.2)	1 (0.9)	1 (1.9)	0 (0.0)	1 (1.2)	1 (3.2)	0 (0.0)	0 (0.0)	1 (3.3)	0 (0.0)	1 (2.0)	0 (0.0)	0 (0.0)
*S. haematobium* + *T. vaginalis* + *N. gonorrhoeae*	1 (0.6)	1 (0.9)	0 (0.0)	0 (0.0)	1 (1.2)	0 (0.0)	0 (0.0)	0 (0.0)	0 (0.0)	1 (1.5)	0 (0.0)	0 (0.0)	0 (0.0)
*S. haematobium* + *T. pallidum*	2 (0.6)	1 (0.9)	0 (0.0)	0 (0.0)	0 (0.0)	0 (0.0)	1 (16.7)	0 (0.0)	1 (3.3)	0 (0.0)	0 (0.0)	0 (0.0)	0 (0.0)
*S. haematobium* + *T. pallidum* + *N. gonorrhoeae*	3 (0.6)	0 (0.0)	1 (1.9)	0 (0.0)	1 (1.2)	0 (0.0)	0 (0.0)	0 (0.0)	1 (3.3)	0 (0.0)	0 (0.0)	0 (0.0)	0 (0.0)
*T. vaginalis + N. gonorrhoeae*	2 (1.2)	2 (1.9)	0 (0.0)	0 (0.0)	1 (1.2)	1 (3.2)	0 (0.0)	0 (0.0)	0 (0.0)	0 (0.0)	2 (4.0)	0 (0.0)	0 (0.0)
*T. pallidum + N. gonorrhoeae*	5 (3.1)	3 (2.9)	2 (3.7)	4 (11.4)	1 (1.2)	0 (0.0)	0 (0.0)	0 (0.0)	1 (3.3)	4 (6.0)	0 (0.0)	0 (0.0)	0 (0.0)

**NON**—no formal education; **CEPE**—*Certificat d’Études Primaires Élémentaires* (primary school certificate); **BEPC**—*Brevet d’Études du Premier Cycle* (lower secondary school certificate); **BACC**—*Baccalauréat* (upper secondary school certificate); **LICENCE/M2**—university-level degree (bachelor’s or master’s level).

**Table 2:** Hematological parameters, age, and BMI in infected vs. non-infected women (n = 159), northern Madagascar, 2024.

Variables	Total (n = 159)	STIs (−) (n = 109)	STIs (+) (n = 50)	*p*-Value	*S. haematobium* (−) (n = 131)	*S. haematobium* (+) (n = 28)	*p*-Value
**Age**				0.959 ^1^			0.889 ^1^
Range	15–80	15–71	18–80		16–80	15–71	
Median	34	34	34		34	33.5	
95%Cl	[35.1–39.3]	[34.6–39.6	[33.6–41.5]		[35.0–39.5]	[31.8–42.8]	
**BMI**				0.840 ^1^			0.791 ^1^
Range	14.4–38.2	15.9–34.1	14.4–38.2		14.4–38.2	16.1–33.0	
Median	21.3	21.3	21.4		21.6	25.8	
95%Cl	[21.3–22.7]	[21.1–22.5]	[20.9–24.0]		[21.2–22.8]	[20.6–23.4]	
**HGB**				0.575 ^1^			0.867 ^1^
Range	5.5–15.1	5.7–14.8	5.5–2		5.5–2	8.7–12.9	
Median	11.5	11.4	11.7		11.5	11.4	
95%Cl	[11.1–11.5]	[11.0–11.6]	[10.8–11.8]		[11.0–11.6]	[10.9–11.7]	
**HTC**				0.271 ^1^			0.566 ^1^
Range	20.6–46.0	21.2–46.0	20.6–45.7		20.6–46	27.6–41.3	
Median	36.5	36.4	37.15		36.9	36.3	
95%Cl	[35.5–36.9]	[35.3–36.8]	[35.0–37.8]		[35.4–37.0]	[34.9–37.2]	
**WBC**				0.012 ^1^			0.269 ^1^
Range	2.2–13.5	2.2–13.5	2.3–12		2.2–13.5	3.6–11.2	
Median	5.6	5.3	6.3		5.6	5.9	
95%Cl	[5.7–6.3]	[5.4–6.1]	[5.9–7.0]		[5.6–6.3]	[5.6–6.9]	
**RBC**				0.845 ^2^			0.636 ^2^
Mean (SD)	4.4	4.4	4.4		4.4	4.4	
Range	2.8–5.6	2.8–5.5	3.2–5.6		2.8–5.6	3.4–5.0	
95%Cl	[4.3–4.5]	[4.3–4.5]	[4.3–4.5]		[4.3–4.5]	[4.2–4.5]	
**MCV**				0.668 ^1^			0.844 ^1^
Range	51.8–95.9	51.8–93.5	58–95.9		51.8–95.9	66.9–93.5	
Median	82.9	83.2	82.5		83.3	82.4	
95%Cl	[81.0–83.3]	[80.6–83.4]	[80.5–84.7]		[80.7–83.4]	[80.5–85.1]	
**MCH**				0.881 ^1^			0.707 ^1^
Range	13.9–30.3	13.9–30.1	15.4–30.3		13.0–30.3	19.9–29.7	
Median	25.8	25.9	25.8		25.9	25.6	
95%Cl	[25.1–26.0]	[25.0–26.1]	[24.7–26.4]		[25.0–26.0]	[25.1–26.7]	
**MCHC**				0.237 ^1^			0.228 ^1^
Range	26.5–31.4	26.5–32.9	26.6–32.8		26.5–32.9	29.7–32.4	
Median	31.2	31.3	31.1		31.2	31.5	
95%Cl	[31.0–31.3]	[31.0–31.4]	[30.6–31.2]		[30.9–31.2]	[31.1–31.6]	
**PLT**				0.179 ^2^			0.108 ^2^
Mean (SD)	248.2	246.8	262.2		255.6	233.3	
Range	107–490	109–372	107–490		107–490	109–372	
95%Cl	241.3–262.0]	[235.0–258.6]	[241.5–282.8]		[244.2–266.9]	[208.1–258.4]	

^1^ Mann–Whitney U test; ^2^ Student’s *t*-test.

## Data Availability

The data presented in this study are available upon request from the corresponding author.

## References

[1] Madagascar (2024). Overview. https://data.worldbank.org/country/madagascar.

[2] Andrianantoandro V.T., Pourette D., Rakotomalala O., Ramaroson H.J.V., Ratovoson R., Rakotoarimanana F.M.J. (2021). Factors influencing maternal healthcare seeking in a highland region of Madagascar: A mixed methods analysis. BMC Pregnancy Childbirth..

[3] Razakamanana M.V., Andrianatoandro V.T., Ramiandrisoa T.O. (2023). Do public health expenditures affect maternal and child health in Madagascar?. Health Econ. Rev..

[4] Chen Y.C., Chayakulkeeree M., Chakrabarti A., Gan G.G., Kwong Y.L., Liu W.-L., Tan B.H., Todi S. (2022). Unmet needs and practical solutions in the management of invasive mould infections in Asia. J. Antimicrob. Chemother..

[5] Institute National de la Statistique (INSTAT) Madagascar DHS, 2021—Final Report (in French). https://dhsprogram.com/publications/publication-FR376-DHS-Final-Reports.cfm.

[6] Rejoice Puthuchira R., Kulasekaran R.A. (2014). Care seeking behaviour and barriers to accessing services for sexual health problems among women in rural areas of Tamilnadu state in India. J. Sex. Transm. Dis..

[7] Robson L., Morris J., Andriatsihosena M. (2015). Barriers to preventing unintended pregnancies and sexually transmitted infections as experienced by women in Fort Dauphin, southeast Madagascar. Eur. J. Contracept. Reprod. Health Care.

[8] Madagascar, Comité National de Lutte Contre le SIDA Rapport D’activité sur la Riposte au SIDA à Madagascar, 2014. https://www.unaids.org/sites/default/files/country/documents/MDG_narrative_report_2014.pdf.

[9] Garchitorena A., Miller A.C., Cordier L.F., Ramananjato R., Rabeza V.R., Murray M., Cripps A., Hall L., Farmer P., Rich M. (2017). In Madagascar, Use Of Health Care Services Increased When Fees Were Removed: Lessons For Universal Health Coverage. Health Aff..

[10] Garchitorena A., Miller A.C., Cordier L.F., Randriamanambintsoa M., Razanadrakato H.R., Randriamihaja M., Razafinjato B., Finnegan K.E., Haruna J., Rakotonirina L. (2020). District-level health system strengthening for universal health coverage: Evidence from a longitudinal cohort study in rural Madagascar, 2014-2018. BMJ Glob. Health.

[11] Schuster A., Randrianasolo B.S., Rabozakandraina O.O., Ramarokoto C.E., Brønnum D., Feldmeier H. (2022). Knowledge, experiences, and practices of women affected by female genital schistosomiasis in rural Madagascar: A qualitative study on disease perception, health impairment and social impact. PLoS Negl. Trop. Dis..

[12] Morris J.L., Short S., Robson L., Andriatsihosena M.S. (2014). Maternal health practices, beliefs and traditions in southeast Madagascar. Afr. J. Reprod. Health.

[13] Institut Pasteur de Madagascar Manuel de Prélèvement des Échantillons Primaires, 2023. https://www.pasteur.mg/wp-content/uploads/2023/11/CBC_MP_001_Manuel_de_pre-levement_des_echantillons_primaires_V14.pdf.

[14] Avni A. (2015). Addressing gender inequalities to improve the sexual and reproductive health and wellbeing of women living with HIV. J. Int. AIDS Soc..

[15] World Health Organization (WHO) (2021). Report on Global Sexually Transmitted Infection Surveillance. https://www.who.int/news-room/fact-sheets/detail/sexually-transmitted-infections-(stis).

[16] World Health Organization (WHO) Global Health Sector Strategy on Sexually Transmitted Infections 2016–2021: Toward ending STIs, 2016. https://www.who.int/publications/i/item/WHO-RHR-16.09.

[17] Mbizvo M.T., Zaidi S. (2010). Addressing critical gaps in achieving universal access to sexual and reproductive health (SRH): The case for improving adolescent SRH, preventing unsafe abortion, and enhancing linkages between SRH and HIV interventions. Int. J. Gynecol. Obstet..

[18] Newton-Levinson A., Leichliter J.S., Chandra-Mouli V. (2017). Help and care seeking for sexually transmitted infections among youth in low- and middle-income countries. Sex. Transm. Dis..

[19] Asres A.W., Endalew M.M., Mengistu S.Y. (2022). Prevalence and trends of sexually transmitted infections among pregnant women in Mizan Tepi University Teaching Hospital, Southwest Ethiopia: A cross-sectional study. Pan Afr. Med. J..

[20] Semwogerere M., Dear N., Tunnage J., Dear N., Tunnage J., Reed D., Kibuuka H., Kiweewa F., Iroezindu M., Bahemana E. (2021). Factors associated with sexually transmitted infections among care-seeking adults in the African Cohort Study. BMC Public Health.

[21] Torrone E.A., Morrison C.S., Chen P.L., Kwok C., Francis S.C., Hayes R.J., Looker K.J., McCormack S., McGrath N., van de Wijgert J.H.H.M. (2018). Prevalence of sexually transmitted infections and bacterial vaginosis among women in sub-Saharan Africa: An individual participant data meta-analysis of 18 HIV prevention studies. PLoS Med..

[22] Shewarega E.S., Fentie E.A., Asmamaw D.B., Negash W.D., Fetene S.M., Teklu R.E., Aragaw F.M., Alemu T.G., Eshetu H.B., Belay D.G. (2022). Sexually transmitted infections related care-seeking behavior and associated factors among reproductive age women in East Africa: A multilevel analysis of demographic and health surveys. BMC Public Health.

[23] Isara A., Baldeh A.K. (2021). Prevalence of sexually transmitted infections among pregnant women attending antenatal clinics in West Coast Region of The Gambia. Afr. Health Sci..

[24] Leutscher P.D., Jensen J.S., Hoffmann S., Berthelsen L., Ramarakoto C.-E., Ramaniraka V., Randrianasolo B., Raharisolo C., Böttiger B., Rousset D. (2005). Sexually transmitted infections in rural Madagascar at an early stage of the HIV epidemic: A 6-month community-based follow-up study. Sex. Transm. Dis..

[25] Behets F., Andriamiadana J., Rasamilalao D., Ratsimbazafy N., Randrianasolo D., Dallabetta G., Cohen M. (2001). Sexually transmitted infections and associated socio-demographic and behavioural factors in women seeking primary care suggest Madagascar’s vulnerability to rapid HIV spread. Trop. Med. Int. Health.

[26] Behets F.M., Andriamiadana J., Randrianasolo D., Rasamilalao D., Ratsimbazafy N., Dallabetta G., Cohen M.S. (2002). Laboratory diagnosis of sexually transmitted infections in women with genital discharge in Madagascar: Implications for primary care. Int. J. STD AIDS..

[27] Frickmann H., Schwarz N.G., Girmann M., Hagen R.M., Poppert S., Crusius S., Podbielski A., Heriniaina J.N., Razafindrabe T., Rakotondrainiarivelo J.P. (2013). Serological survey of HIV and syphilis in pregnant women in Madagascar. Trop. Med. Int. Health.

[28] Xueref S., Holianjavony J., Daniel R., Kerouedan D., Fabry J., Vanhems P. (2003). The absence of HIV seropositivity contrasts with a high prevalence of markers of sexually transmitted infections among registered female sex workers in Toliary, Madagascar. Trop. Med. Int. Health.

[29] Banque Mondiale Programme Global de Lutte Contre le VIH/SIDA. VIH/SIDA—Obtenir des Résultats, 2008. http://siteresources.worldbank.org/INTHIVAIDS/Resources/375798-1132695455908/M&EGRMadagascarFR.pdf.

[30] Fortas C., Harimanana A.N., Rasoanandrianina S.B., Rasoanaivo T.F., Razanadranaivo H.L., Mangahasimbola R.T., Rasolon D.T., Rafetrarivony L.F., Rasolofomanana T.T., Rabarisoa L. (2025). Sexually transmitted infections and bacterial vaginosis in women of child-bearing age in Antananarivo, Madagascar: Prevalence and risk factors from a cross-sectional study. BMC Infect. Dis..

[31] Bigoni J., Catarino R., Benski C., Viviano M., Munoz M., Tilahizandry H., Petignat P., Vassilakos P. (2018). High Burden of Human Papillomavirus Infection in Madagascar: Comparison With Other Sexually Transmitted Infections. Infect. Dis. Res. Treat..

[32] Centers for Disease Control and Prevention (2023). Sexually Transmitted Infections in Developing Countries. http://web.worldbank.org/archive/website01213/WEB/IMAGES/AAGSTIFI.PDF.

[33] Sullivan P.S., Hanson D.L., Chu S.Y., Jones J.L., Ward J.W. (1998). Epidemiology of anemia in human immunodeficiency virus (HIV)-infected persons: Results from the multistate adult and adolescent spectrum of HIV disease surveillance project. Blood.

[34] Adapen C., Réot L., Menu E. (2022). Role of the human vaginal microbiota in the regulation of inflammation and sexually transmitted infection acquisition: Contribution of the non-human primate model to a better understanding?. Front. Reprod. Health.

[35] Leutscher P.D., Ramarokoto C.E., Hoffmann S., Jensen J.S., Ramaniraka V., Randrianasolo B., Raharisolo C., Migliani R., Christensen N. (2008). Coexistence of urogenital schistosomiasis and sexually transmitted infection in women and men living in an area where Schistosoma haematobium is endemic. Clin. Infect. Dis..

[36] Gruninger S.K., Rasamoelina T.R., Rakotoarivelo R.A., Razafindrakoto A.R., Rasolojaona Z.T., Rakotozafy R.M., Soloniaina P.R., Rakotozandrindrainy N., Rausche P., Doumbia C.O. (2023). Prevalence and risk distribution of schistosomiasis among adults in Madagascar: A cross-sectional study. Infect. Dis. Poverty.

[37] Rollinson D., Knopp S., Levitz S., Stothard J.R., Tchuem Tchuenté L.A., Garba A., Mohammed K.A., Schur N., Person B., Colley D.G. (2013). Time to set the agenda for schistosomiasis elimination. Acta Trop..

[38] Hotez P.J., Engels D., Gyapong M., Ducker C., Malecela M.N. (2019). Female genital schistosomiasis. N. Engl. J. Med..

[39] Patel P., Rose C.E., Kjetland E.F., Downs J.A., Mbabazi P.S., Sabin K., Chege W., Watts D.H., Secor W.E. (2021). Association of schistosomiasis and HIV infections: A systematic review and meta-analysis. Int. J. Infect. Dis..

[40] Kutz J.M., Rausche P., Rasamoelina T., Ratefiarisoa S., Razafindrakoto R., Klein P., Jaeger A., Rakotomalala R.S., Rakotomalala Z., Randrianasolo B.S. (2023). Female genital schistosomiasis, human papilloma virus infection, and cervical cancer in rural Madagascar: A cross sectional study. Infect. Dis. Poverty.

[41] Mazigo H.D., Samson A., Lambert V.J., Kosia A.L., Ngoma D.D., Murphy R., Matungwa D.J. (2021). “We know about schistosomiasis but we know nothing about FGS”: A qualitative assessment of knowledge gaps about female genital schistosomiasis among communities living in Schistosoma haematobium endemic districts of Zanzibar and northwestern Tanzania. PLoS Negl. Trop. Dis..

[42] Rausche P., Rakotoarivelo R.A., Rakotozandrindrainy R., Rakotomalala R.S., Ratefiarisoa S., Rasamoelina T., Kutz J.-M., Jaeger A., Hoeppner Y., Lorenz E. (2023). Awareness and knowledge of female genital schistosomiasis in a population with high endemicity: A cross-sectional study in Madagascar. Front. Microbiol..

[43] Angora E.K., Boissier J., Menan H., Rey O., Tuo K., Touré A.O., Coulibaly J.T., Méité A., Raso G., N’Goran E.K. (2019). Prevalence and Risk Factors for Schistosomiasis among Schoolchildren in two Settings of Côte d’Ivoire. Trop. Med. Infect. Dis..

[44] Huang Q., Gurarie D., Ndeffo-Mbah M., Li E., King C.H. (2022). Schistosoma Transmission in a Dynamic Seasonal Environment and its Impact on the Effectiveness of Disease Control. J. Infect. Dis..

[45] Rakotomamonjy L., Andréambeloson T., Randriamihaja M., Ihantamalala F., Cordier L., Cowley G., Finnegan K., Hanitriniaina F., Miller A.C., Marovavy L. (2020). Healthcare Access in Rural Madagascar: A Study of Barriers in the Tsimihety Region. Health Policy Plan..

